# Are There Any Differences in Clinical and Biochemical Variables between Bipolar Patients with or without Lifetime Psychotic Symptoms?

**DOI:** 10.3390/jcm12185902

**Published:** 2023-09-11

**Authors:** Cecilia Maria Esposito, Jennifer L. Barkin, Alessandro Ceresa, Guido Nosari, Martina Di Paolo, Francesca Legnani, Luisa Cirella, Teresa Surace, Ilaria Tagliabue, Enrico Capuzzi, Alice Caldiroli, Antonios Dakanalis, Pierluigi Politi, Massimo Clerici, Massimiliano Buoli

**Affiliations:** 1 Department of Neurosciences and Mental Health, Fondazione IRCCS Ca’ Granda, Ospedale Maggiore Policlinico, 20122 Milan, Italy; alessandro.ceresa@unimi.it (A.C.); guido.nosari@unimi.it (G.N.); martina.dipaolo@unimi.it (M.D.P.); francesca.legnani@unimi.it (F.L.); 2 Department of Brain and Behavioral Sciences, University of Pavia, 27100 Pavia, Italy; pierluigi.politi@unipv.it; 3 Department of Community Medicine, School of Medicine, Mercer University, Macon, GA 31207, USA; barkin_jl@mercer.edu; 4 Healthcare Professionals Department, Foundation IRCCS Ca’ Granda Ospedale Maggiore Policlinico, 20122 Milan, Italy; luisa_cirella@policlinico.mi.it; 5 Psychiatric Department, Azienda Socio Sanitaria Territoriale Monza, 20900 Monza, Italy; teresa.surace70@gmail.com (T.S.); i.tagliabue5@campus.unimib.it (I.T.); e.capuzzi1@campus.unimib.it (E.C.); alyscaldi@gmail.com (A.C.); massimo.clerici@unimib.it (M.C.); 6 Department of Medicine and Surgery, University of Milano Bicocca, 20126 Monza, Italy; antonios.dakanalis@unimib.it; 7 Department of Pathophysiology and Transplantation, University of Milan, 20122 Milan, Italy

**Keywords:** bipolar disorder (BD), lifetime psychotic symptoms, clinical variables, biochemical parameters

## Abstract

Introduction: Psychotic symptoms occur in more than half of patients affected by Bipolar Disorder (BD) and are associated with an unfavorable course of the disorder. The objective of this study is to identify the differences in the clinical and biochemical parameters between bipolar patients with or without psychotic symptoms. Methods: A total of 665 inpatients were recruited. Demographic, clinical, and biochemical data related to the first day of hospitalization were obtained via a screening of the clinical charts and intranet hospital applications. The two groups identified via the lifetime presence of psychotic symptoms were compared using t tests for quantitative variables and χ^2^ tests for qualitative ones; binary logistic regression models were subsequently performed. Results: Patients with psychotic BD (compared to non-psychotic ones) showed a longer duration of hospitalization (*p* < 0.001), higher Young Mania Rating Scale scores (*p* < 0.001), lower Global Assessment of Functioning scores (*p* = 0.002), a less frequent history of lifetime suicide attempts (*p* = 0.019), less achievement of remission during the current hospitalization (*p* = 0.028), and a higher Neutrophile to Lymphocyte Ratio (NLR) (*p* = 0.006), but lower total cholesterol (*p* = 0.018) and triglycerides (*p* = 0.013). Conclusions: Patients with psychotic BD have a different clinical and biochemical profile compared to their counterparts, characterized by more clinical severity, fewer metabolic alterations, and a higher grade of inflammation. Further multi-center studies have to confirm the results of this present study.

## 1. Introduction

Bipolar disorder (BD) is a chronic psychiatric condition characterized by the recurrence of hypomanic/manic and depressive episodes, with inter-episodic euthymia [1]. Depending on the presence of manic episodes, it is possible to identify BD type 1, which is associated with less favorable clinical profile and more suicide attempts [2], and BD type 2, which is not often promptly diagnosed for the predominance of depressive symptoms [2,3,4]. According to the literature, more than 1% of the global population is affected by BD, with consequently a prominent economic impact on the health systems [5,6]. Several studies highlighted that a long duration of untreated illness has a negative impact on the course of BD, being associated with a higher risk of suicide attempts and hospitalizations [7,8,9]. Of note, if not promptly treated, BD significantly reduces life expectancy by nearly 10–20 years [10]. The BD-associated disability can be attributed to the severity and duration of the episodes, hospitalizations, chronicity, cognitive impairment, and the impact of the illness on psycho-social functioning [11,12,13]. 

Psychotic symptoms (hallucinations and delusions) can occur during both depressive and manic episodes but are more prevalent during mania [14]. Of note, available literature indicates that psychotic features can occur in up to 90% of BD type 1 patients and 50% of BD type 2 patients lifetime [14]. It must also be considered that the presence of psychotic symptoms often complicates the course of BD as a result of misdiagnosis and delayed proper management of patients: BD with psychotic symptoms can be confused with other psychotic disorders, such as schizophrenia or substance-induced psychotic disorder [15,16]. Moreover, the presence of psychotic symptoms complicates the course of illness; the previous literature highlighted that delusions and hallucinations are associated with mixed affective episodes, poor functional outcomes, and suicidal behavior [17,18,19]. Furthermore, psychotic bipolar patients compared to their counterparts show generally a more compromised general health and more medical comorbidity [20].

In light of the impact that psychotic symptoms can have on the course of BD, biomarkers that help to discriminate between BD with or without psychotic symptoms may be of great clinical utility [21]. Several biomarkers were tested in their ability to discriminate psychotic versus non-psychotic BD, and the differences in several biological systems were identified between these two groups of patients [22]. Regarding genetics, some authors explored the possibility that polymorphisms of genes involved in the regulation of neurotransmission (e.g., *Catechol-O-methyltransferase-COMT*) [23] or neuron function (e.g., *Neuroregulin 1-NRG1*) confer vulnerability to develop psychotic symptoms in BD [24]. In addition, over-inflammation or hormonal dysregulation was found to be more prominent in bipolar individuals with versus without psychotic features [25]. The preliminary findings from neuroimaging studies confirmed that psychotic bipolar patients resulted in more brain alterations than non-psychotic ones, such as more atrophy in the angular gyrus or less white matter integrity [26,27]. In line with the results of neuroimaging studies, social cognition seems to be more compromised in psychotic BD than in bipolar patients without psychotic symptoms [28]. 

Only recently, some investigators directed their research to identify potential biochemical parameters associated with specific clinical features of BD. Of note, a recent article found that bipolar patients with lifetime suicide attempts presented lower bilirubin plasma levels than those without suicidal behavior [29]. In addition, the presence of psychotic symptoms in mood disorders would seem to be associated with lower vitamin E plasma levels [30]. 

Given the scarcity of data in this area, the main objective of this present study is to identify clinical variables and biochemical parameters associated with the presence of psychotic symptoms in BD to provide potential ideas for the management of these patients.

## 2. Methods

### 2.1. Participants and Study Design

For this retrospective study, a total sample of 655 patients with a diagnosis of BD was collected from those consecutively hospitalized between 2002 and 2022 in inpatient clinics of ASST Monza, Italy (N = 149) or Fondazione IRCCS Policlinico (N = 516). All patients were screened by an expert senior psychiatrist through a clinical interview, and they met the criteria for the diagnosis of BD according to DSM criteria [1]. Per DSM criteria, psychotic bipolar patients were differentiated from subjects suffering from schizoaffective disorders since the latter persist in having psychotic symptoms after the remission of mood episodes [1]. If they met the criteria for other psychiatric diagnoses, BD was still the most clinically relevant diagnosis (associated with more social dysfunction). Clinical and biochemical data were retrospectively obtained via screening of the clinical charts and intranet hospital applications. In these inpatient clinics, it is used to conduct routine blood analyses during the first day of hospitalization in the morning. At the end of the hospitalization, after being discharged, these patients are generally long-term followed-up in the community mental health services pertaining to the clinics mentioned above.

### 2.2. Variables and Assessment Instruments

The following variables were collected at the admission to the hospital:

- Demographic and clinical variables: age, gender, age at onset, duration of untreated illness in years (defined as the time between the onset of the disorder and the prescription of an adequate treatment) [8], duration of illness (years), duration of the current hospitalization (days), number of lifetime hospitalizations, number of lifetime mood episodes, type of current episode (mania or major depressive episode), type of last episode (manic, hypomanic, depressive episodes), current mixed features, lifetime history of substance or alcohol use disorders, presence of lifetime history of poly-substance use disorders, lifetime history of mixed features, lifetime history of psychotic symptoms, lifetime presence of rapid cycles, lifetime presence of seasonality, family history for single or multiple psychiatric disorders, main treatment at the time of hospitalization, poly-therapy at the time of admission, psychiatric comorbidity, comorbidity with personality disorders, type of personality disorder, presence of lifetime suicidal attempts (defined as self-harm combined with the intent to die) [31], number of lifetime suicidal attempts, current smoking status, number of cigarettes/day, number of depressive episodes lifetime and in the last year, number of manic episodes lifetime and in the last year, number of hypomanic episodes lifetime and in the last year, number of total mood episodes lifetime and in the last year, number of lifetime episodes induced from substance abuse, obstetric complications, presence of a medical comorbidity, comorbidity with thyroid disorders, comorbidity with diabetes, comorbidity with obesity (defined as a body mass index (BMI) ≥ 30) [31], BMI, comorbidity with hypercholesterolemia, presence of multiple medical comorbidities, current treatment with statin, current treatment with levotiroxine, response to therapy in the last episode (defined as a reduction of at least 50% of the baseline total rating scale scores) [32,33], remission in the last episode (defined as an endpoint HAM-D score < 8 and a YMRS score < 10) [32,34], and treatment response and remission during the current hospitalization, and scores of Young Mania Rating Scale (YMRS) [33], Hamilton Depression Rating Scale (HAM-D) [35], Montgomery and Åsberg Depression Rating Scale (MADRS) [36], Hamilton Anxiety Rating Scale (HAM-A) [37], and Global Assessment of Functioning (GAF) [38].
- Biochemical parameters: number of red blood cells (RBC) (10^12^/L), mean corpuscular volume (MCV) (fL), hemoglobin (HB) (g/dL), number of white blood cells (WBC) (10^9^/L), number of lymphocytes (10^9^/L), number of neutrophils (10^9^/L), neutrophil/lymphocyte ratio (NLR), number of platelets (10^9^/L), mean platelet volume (MPV) (fL), pseudocholinesterase (PCHE) (U/L), total plasmatic proteins (g/dL), albumin (g/dL), bilirubin (mg/dL), creatinine (mg/dL), uric acid (mg/dL), cholesterol (mg/dL), low-density lipoproteins (LDL) (mg/dL), high-density lipoproteins (HDL) (mg/dL), glycaemia (mg/dL), urea (mg/dL), creatine phosphokinase (CPK) (U/L), thyroid-stimulating hormone (TSH) (mcU/mL), transaminases (aspartate transaminase-AST and alanine transaminases-ALT) (UI/L), gamma-glutamyl-transferase (GGT) (U/L), lactate dehydrogenase (LDH) (mU/mL), triglycerides (mg/dL), serum iron (mcg/dL), C-reactive protein (mg/L), sodium (mmol/L), potassium (mmol/L), and sodium/potassium ratio (Na/K ratio). The biochemical parameters were selected in line with previous studies by our research group [29,39].

### 2.3. Primary Outcomes

The primary outcome of this present study is to identify clinical characteristics and biochemical features capable of differentiating between bipolar patients with and without psychotic patients. This study was conducted in accordance with the provisions of the Declaration of Helsinki, and the protocol was approved by the local Ethical Committee (Fondazione IRCCS Ca’ Granda Ospedale Maggiore Policlinico) (approval number 1789). 

### 2.4. Inclusion and Exclusion Criteria

The following inclusion criteria were used to define the sample: (1) age ≥ 18 years, (2) admission to inpatient clinics between 2002 and 2021, and (3) diagnosis of BD. For patients who had multiple hospitalizations, we took into account only the last one.

Moreover, the following exclusion criteria were set: (1) age ≤ 18 years, (2) pharmacological treatments that could favor the onset of psychotic symptoms (e.g., steroids and levetiracetam); (3) medical comorbidities that could significantly affect biochemical parameters (e.g., rheumatoid arthritis); (4) peripartum (pregnancy to one month after delivery) as this period is characterized by biological changes in women and susceptibility to the exacerbation of psychiatric symptoms [40].

### 2.5. Statistical Analyses

Statistical analyses were performed using The Statistical Package for Social Sciences (SPSS) for Windows (version 27.0). First of all, descriptive analyses of the whole sample were performed. Then, the total sample was divided into two groups according to the presence of lifetime psychotic symptoms (psychotic/non-psychotic BD), and the two groups were compared using independent sample Student’s *t* tests for continuous variables and chi-square tests for categorical variables (with odds ratio [OR] and 95% confidence interval [CI] calculation where appropriate). Those variables that proved to be statistically significant from these preliminary analyses were inserted in binary logistic regression models (enter method), considering the presence of lifetime psychotic symptoms as dependent variable. We computed three different binary logistic regression analyses, two for clinical variables (one for lifetime variables and one for current/last year clinical factors) and one for biochemical parameters. In the end, every statistically significant variable from the three above-mentioned binary logistic regression models was inserted in a new global multivariable logistic regression model (enter method). A similar statistical approach was used by our group in other analyses on different samples due to the supposed large number of variables statistically related to the dependent variable (in this case psychotic symptoms) [40]. The goodness of the models was assessed using the Omnibus and Hosmer–Lemeshow tests. The level of statistical significance was set at *p* ≤ 0.05. 

## 3. Results

Among the total sample of 665 patients, 236 (35.5%) did not manifest lifetime psychotic symptoms, while the remaining 429 (64.5%) experienced this type of symptoms. The total sample was composed of 391 (58.8%) females and 274 (41.2%) males, and patients had a mean age of 46.46 (±14.25) years at the time of hospitalization. 

Data about clinical variables of the total sample and of the two groups are provided in Table 1, while those for biological variables are reported in Table 2. Information about the main pharmacological treatment at the time of hospitalization was available for 385 patients: 85 (22.1%) were drug free, 134 (34.8%) were receiving a mood stabilizer (16.9% lithium, 16.1% valproic acid, 1.0% carbamazepine, 0.8% lamotrigine), 90 (23.4%) an antipsychotic treatment (6.2% olanzapine, 6.0% quetiapine, 4.2% haloperidol, 2.9% aripiprazole, 1.6% paliperidone, 1.3% zuclopenthixol, 0.8% risperidone, 0.2% asenapine, 0.2% lurasidone), 73 (18.9%) an antidepressant treatment (8.1% Selective Serotonin Reuptake Inhibitors-SSRIs, 5.7% Serotonin and Norepinephrine Reuptake Inhibitors-SNRIs, 2.0% tricyclic antidepressants, 1.3% vortioxetine, 1.0% mirtazapine, 0.8% trazodone), and finally 3 (0.8%) gabapentin. Non-psychotic bipolar patients had been more frequently treated with tricyclic antidepressants, SNRIs, and gabapentin compared to their counterparts (all *p* < 0.05).

### 3.1. Clinical Variables

At univariate analyses psychotic versus non-psychotic bipolar patients resulted in the following: more represented in the male gender (χ^2^ = 4.061, *p* = 0.044, OR: 1.398 [1.009–1.942]), younger (t = 3.081, *p* = 0.002), have a shorter duration of illness (t = 2.222, *p* = 0.027), more frequently hospitalized for a manic episode (χ^2^ = 11.380, *p* = 0.001, OR: 1.786 [1.295–2.688]), a longer duration of hospitalization (t = 5.170; *p* < 0.001), more frequently suffered from a previous major depressive episode (χ^2^ = 19.393, *p* < 0.001), smoke less cigarettes/day (t = 2.537, *p* = 0.012), more lifetime manic episodes (t = 3.735, *p* < 0.001), less lifetime hypomanic episodes (t = 2.032, *p* = 0.043), less depressive episodes in the last year (t = 1.999, *p* = 0.047), present less often current mixed features (χ^2^ = 6.902, *p* = 0.009, OR: 0.630 [0.446–0.890]), less frequently a comorbidity with a personality disorder (χ^2^ = 5.702, *p* = 0.017, OR: 0.524 [0.306–0.896]), less frequently a history of lifetime suicidal attempts (χ^2^ = 5.490, *p* = 0.019, OR: 0.604 [0.395–0.923]), less often a comorbidity with multiple medical disorders (χ^2^ = 3.871, *p* = 0.049, OR: 0.647 [0.419–0.999]), achieve less frequently clinical remission during the current hospitalization (χ^2^ = 4.807, *p* = 0.028, OR: 0.638 [0.427–0.955]), and show less GAF scores (t = 3.157, *p* = 0.002) and higher YMRS (t = 5.685, *p* < 0.001) and BPRS scores (t = 7.588, *p* < 0.001).

With regard to the preliminary binary logistic regression with lifetime clinical variables, the goodness-of-fit test (Hosmer and Lemeshow Test: χ^2^ = 4.329, *p* = 0.826) showed that the model was reliable, allowing for a correct classification of 66.8% of cases. In addition, the model was overall significant (Omnibus test: χ^2^ = 32.646, df = 11, *p* = 0.001). Patients with a history of psychotic symptoms resulted to have more lifetime manic episodes (*p* = 0.047) and less frequently a comorbid personality disorder at borderline statistical significance (*p* = 0.057) than their counterparts. Regarding the preliminary logistic regression with recent/last year clinical variables, the model resulted to be reliable (Hosmer and Lemeshow Test: χ^2^ = 8.868, *p* = 0.354; Omnibus test: χ^2^ = 57.612, df = 9, *p* = 0.001), allowing for a correct classification of 73% of cases. Patients with a history of psychotic symptoms resulted in a longer hospitalization (*p* = 0.014), higher YMRS scores (*p* = 0.007), and more frequent current hospitalization for a manic episode than the counterpart (*p* = 0.014). 

### 3.2. Biological Parameters

Regarding biological variables, psychotic versus non-psychotic bipolar patients resulted in a higher NLR (t = −2.776, *p* = 0.006) and higher bilirubin (t = 2.348, *p* = 0.019) and CPK plasma levels (t = 2.807, *p* = 0.005). In contrast, patients with a psychotic BD showed lower cholesterol (t = 2.369; *p* = 0.018), GGT (t = 2.249; *p* = 0.026), and triglyceride (t = 2.554; *p* = 0.013) plasma levels than their counterparts. In addition, the model of preliminary binary logistic regression analysis with biochemical parameters resulted to be reliable (Hosmer and Lemeshow Test: χ^2^ = 21.884, *p* = 0.003; Omnibus test: χ^2^ = 14.524, df = 7, *p* = 0.043), allowing for a correct classification of 89.2% of cases. Only NLR was shown to predict the presence of lifetime psychotic symptoms in BD (*p* = 0.024).

### 3.3. Final Binary Logistic Regression Model

The final binary logistic regression model was reliable (Hosmer and Lemeshow Test: χ^2^ = 8.024, *p* = 0.431; Omnibus test: χ^2^ = 47.808, df = 6, *p* < 0.001), allowing for a correct classification of 70.9% of cases. The duration of hospitalization (*p* = 0.007), higher YMRS scores (*p* < 0.001), as well as a manic episode (*p* = 0.001) as reasons for hospitalization were confirmed to be significantly associated with lifetime presence of psychotic symptoms in BD (Table 3).

## 4. Discussion

The findings of this present research confirm that psychotic bipolar patients present a clinical profile different from that of non-psychotic bipolar subjects [41]. Globally, the presence of psychotic symptoms in BD was found to be associated with unfavorable clinical features such as a longer hospital stay [42], less social functioning (GAF scores) [43], and less probability of achieving clinical remission during hospitalization [14] in agreement with previous reports. Different factors can explain the greater difficulty in obtaining clinical stabilization of psychotic versus non-psychotic bipolar patients, including more severity of illness at the time of admission to the hospital [44,45,46], the negative impact of the more numerous previous manic episodes [15,47,48] and the predominance of male gender that is more frequently characterized by comorbid substance use disorders [49]. Of note, some authors argued that bipolar women can have a better response to pharmacotherapy compared to men [50]. In addition, male hormones would favor the onset of psychotic symptoms in patients suffering from mood disorders [51], as revealed in previous studies, which showed that how higher dehydroepiandrosterone sulfate plasma levels could be associated with the presence of lifetime psychotic symptoms [52,53]. Furthermore, bipolar patients experiencing psychotic symptoms have a shorter duration of illness, probably indicating a severe clinical presentation requiring hospitalization already in the early phases of BD [54,55]. On the contrary, the lower presence of male gender and of current mixed features, as well as more depressive episodes in non-psychotic bipolar subjects, would explain the higher frequency of suicide attempts in this group than in psychotic bipolar patients. Of note, previous research repeatedly reported that female gender [56], depressive episodes [57], and mixed features [58] are associated with a higher risk of suicidal attempts in BD. Finally, the younger age of psychotic bipolar patients versus the counterpart would reasonably explain in this group the lower frequency of multiple medical comorbidity that typically characterizes chronic bipolar patients as a result of unhealthy lifestyles or poor adherence to medical prevention programs, among other factors [59]. 

Concerning biological variables, to our knowledge, this is the first research that found that psychotic bipolar patients have higher NLR plasma levels compared to their counterparts. This result can be explained by the fact that patients with lifetime psychotic symptoms experience more manic episodes and have globally more severe manic symptoms at hospital admission. Of note, different authors demonstrated not only that NLR is more elevated in bipolar patients than healthy subjects but also that manic episodes are associated with higher levels of this parameter [60,61]. Another possible explanation is that psychotic BD can be seen as an intermediate phenotype between non-psychotic BD and schizophrenia; in support of this assertion, higher NLR was found in schizophrenia versus bipolar patients [62]. On the other hand, the mean NLR values of our total sample are outside the range calculated in healthy populations, indicating that BD is characterized by low-grade systemic inflammation [63]. A higher grade of inflammation in psychotic versus non-psychotic bipolar patients is also supported by the lower GGT plasma levels in the first group, as this enzyme has antioxidant properties [64]. 

On the contrary, metabolic parameters (total cholesterol and triglycerides) were higher in patients with non-psychotic BD than in patients with the psychotic subtype. This finding can be explained by differences in diet between the two groups: patients without psychotic symptoms might consume more alcohol as they experience more recent depressive episodes and current mixed episodes than their counterparts. Of note, more depressive episodes and less severe mania were reported to be associated with lifetime alcohol consumption [65]. This is also in line with our finding of higher GGT in non-psychotic versus psychotic bipolar patients [64]. In addition, several authors reported that the severity of depressive symptoms is proportionally related to cholesterol plasma mean levels [53,66]. An alternative explanation can be the over-representation of the female gender in the non-psychotic group because women seem more susceptible to the development of metabolic disorders in the case of mood [40,67] and psychotic disorders [67,68]. 

Some authors reported that unconjugated bilirubin is higher in schizophrenia than in BD, in agreement with our data and supporting the hypothesis that psychotic BD would represent an intermediate phenotype [69,70]. Finally, more CPK elevation in manic versus depressive episodes was previously reported, in agreement with our results that outlined higher CPK in psychotic versus non-psychotic patients as a result of more lifetime manic episodes [71]. 

The following limitations must be listed for this present study: (1) the last treatment with different psychotropic drugs may have influenced clinical and biological parameters, but only for some antidepressants and gabapentin, a significant difference in the rate of prescription was identified in the two groups; (2) the presence of substance use disorders or treatments for medical comorbidities may have affected the assessed variables; (3) the retrospective design implying that the information derived from clinical charts or patients’ interviews may be not always accurate; (4) the use of routinely investigated biochemical parameters during hospital admissions, without a preliminary definition; (5) a lot of data are missing because some parameters are not routinely collected at the admission of patients in one or both centers; and (6) the sample limited to the catchment area of our departments.

Further studies are needed to confirm that psychotic versus non-psychotic bipolar subjects have different clinical and biological profiles. With regard to biological parameters, also drug-free samples can be useful to avoid the impact of pharmacological treatment on these variables. Moreover, the relationship between clinical features and biochemical parameters should be better clarified, such as the complex interplay of cholesterol levels and its hormonal derivative vitamin D with the severity/quality of psychiatric symptoms [72,73]. These future directions of research will allow for promoting personalized treatment and implementing specific preventive strategies.

## Figures and Tables

**Table 1:** Demographic and clinical variables of the total sample and of the two groups divided according to the presence of lifetime psychotic symptoms.

Variables			Total Sample N = 665	−Psychotic Symptoms N = 236 (35.5%)	+Psychotic Symptoms N = 429 (64.5%)	χ^2^ or t	*p*-Value
Gender (Missing = 0)	Male		274 (41.2%)	85 (36.0%)	189 (44.1%)	4.061	**0.044**
	Female		391 (58.8%)	151 (64.0%)	240 (55.9%)		
Age (years) (Missing = 0)			46.46 (±14.25)	48.75 (±13.91)	45.21 (±14.30)	3.081	**0.002**
Age at onset (years) (Missing = 97)			28.97 (±11.23)	29.93 (±12.06)	28.46 (±10.75)	1.490	0.137
Duration of untreated illness (years) (Missing = 243)			3.03 (±5.41)	3.48 (±5.71)	2.75 (±5.21)	1.336	0.182
Duration of illness (years) (Missing = 97)			17.69 (±12.90)	19.33 (±13.16)	16.81 (±12.69)	2.222	**0.027**
Type of current episode		Mania	509 (76.5%)	163 (69.1%)	346 (80.1%)	11.380	**0.001**
		Major Depression	156 (23.5%)	73 (30.9%)	83 (19.9%)		
Duration of current hospitalization (days) (Missing = 150)			12.74 (±8.31)	10.56 (±5.58)	13.95 (±9.29)	5.170	**<0.001**
Type of last episode (Missing = 306)	No previous episodes (current first episode)		3 (0.8%)	1 (0.7%)	2 (0.9%)	19.393	**<0.001**
	Major Depression		150 (41.8%)	37 (27.2%)	113 (50.7%)		
	Mania		150 (41.8%)	71 (52.2%)	79 (35.4%)		
	Hypomania		56 (15.6%)	27 (19.9%)	29 (13.0%)		
Number of cigarettes/day (Missing = 344)			9.40 (±12.11)	11.71 (±12.73)	8.15 (±11.59)	2.537	**0.012**
Number of lifetime hospitalizations (Missing = 148)			3.34 (±4.53)	3.01 (±4.59)	3.51 (±4.50)	1.174	0.241
Number of lifetime mood episodes (Missing = 198)			6.06 (±5.37)	5.76 (±5.55)	6.22 (±5.28)	0.886	0.376
Number of lifetime manic episodes (Missing = 197)			2.49 (±3.35)	1.71 (±3.62)	2.90 (±3.13)	3.735	**<0.001**
Number of lifetime hypomanic episodes (Missing = 198)			1.34 (±1.91)	1.58 (±2.02)	1.20 (±1.83)	2.032	**0.043**
Number of lifetime depressive episodes (Missing = 199)			2.22 (±2.02)	2.45 (±1.99)	2.09 (±2.03)	1.805	0.072
Number of depressive episodes in the last year (Missing = 229)			0.32 (±0.63)	0.41 (±0.74)	0.27 (±0.56)	1.999	**0.047**
Number of manic episodes in the last year (Missing = 238)			0.99 (±0.67)	0.98 (±0.69)	0.99 (±0.66)	0.128	0.898
Number of hypomanic episodes in the last year (Missing = 237)			0.21 (±0.57)	0.25 (±0.63)	0.18 (±0.53)	1.210	0.227
Number of total episodes in the last year (Missing = 237)			1.48 (±1.01)	1.58 (±1.09)	1.43 (±0.96)	1.071	0.134
Number of lifetime episodes induced by substances of abuse (Missing = 282)			0.13 (±0.46)	0.10 (±0.36)	0.15 (±0.51)	1.071	0.285
Lifetime presence of rapid cycles (Missing = 202)			63 (13.6%)	23 (13.8%)	40 (13.5%)	0.006	0.938
Lifetime presence of seasonality (Missing = 263)			28 (7.0%)	11 (7.4%)	17 (6.7%)	0.064	0.801
Current mixed features (Missing = 3)			189 (28.5%)	82 (34.7%)	107 (25.1%)	6.902	**0.009**
Lifetime history of mixed features (Missing = 229)			244 (56.0%)	78 (51.7%)	166 (58.3%)	1.739	0.187
Lifetime history of substance use disorders (Missing = 59)			194 (32.0%)	69 (31.2%)	125 (32.5%)	0.100	0.752
Lifetime history of alcohol misuse (Missing = 78)			114 (19.4%)	50 (23.0%)	64 (17.3%)	2.884	0.089
Lifetime history of poly-substance use disorders (Missing = 70)			76 (12.8%)	25 (11.5%)	51 (13.5%)	0.481	0.488
Family history of psychiatric disorders (Missing = 257)			201 (49.3%)	77 (52.0%)	124 (47.7%)	0.709	0.400
Family history of multiple psychiatric disorders (Missing = 268)			121 (30.5%)	42 (29.2%)	79 (31.2%)	0.184	0.668
Current smoking habit (Missing = 225)			241 (54.8%)	90 (58.8%)	151 (52.6%)	1.554	0.213
Therapy with more than one drug at the time of hospitalization (Missing = 267)			170 (42.7%)	64 (44.1%)	106 (41.9%)	0.189	0.664
Comorbidity with a psychiatric diagnosis (Missing = 481)	No comorbidity		107 (58.2%)	39 (54.2%)	68 (60.7%)	5.876	0.209
	Eating disorders		10 (5.4%)	2 (2.8%)	8 (7.1%)		
	GAD		55 (29.9%)	24 (33.3%)	31 (27.7%)		
	Social Anxiety		5 (2.7%)	4 (5.6%)	1 (0.9%)		
	OCD		7 (3.8%)	3 (4.2%)	4 (3.6%)		
Comorbidity with a diagnosis of personality disorder (Missing = 184)			62 (12.9%)	31 (17.7%)	31 (10.1%)	5.702	**0.017**
Presence of history of lifetime suicidal attempts (Missing = 152)			114 (22.2%)	51 (28.0%)	63 (19.0%)	5.490	**0.019**
Number of lifetime suicide attempts (Missing = 160)			0.37 (±0.87)	0.46 (±0.99)	0.32 (±0.83)	1.666	0.097
Presence of comorbidity with a medical condition (Missing = 229)			187 (42.9%)	72 (45.6%)	115 (41.4%)	5.510	0.788
Obstetric complication (Missing = 187)			9 (1.9%)	2 (1.1%)	7 (2.3%)	0.818	0.366
Comorbidity with thyroid disorders (Missing = 171)			78 (15.8%)	36 (19.8%)	42 (13.5%)	3.452	0.063
Comorbidity with diabetes (Missing = 172)			45 (9.1%)	18 (9.8%)	27 (8.7%)	0.176	0.675
Comorbidity with hyper-cholesterolemia (Missing = 234)			106 (24.6%)	41 (25.8%)	65 (23.9%)	0.193	0.660
Comorbidity with multiple medical disorders (Missing = 245)			118 (28.1%)	52 (33.8%)	66 (24.8%)	3.871	**0.049**
Comorbidity with obesity (Missing = 259)			31 (7.6%)	11 (7.2%)	20 (7.9%)	0.055	0.815
BMI (Missing = 517)			25.10 (±5.42)	25.62 (±5.50)	24.80 (±5.38)	0.880	0.380
Response to therapy in the last episode (Missing = 561)			100 (96.2%)	32 (100.0%)	68 (94.4%)	1.849	0.174
Current treatment response (Missing = 160)			470 (93.1%)	173 (95.6%)	297 (91.7%)	2.757	0.097
Remission in the last episode (Missing = 557)			73 (67.6%)	25 (71.4%)	48 (65.8%)	0.248	0.555
Current remission (Missing = 160)			344 (68.1%)	135 (74.2%)	209 (64.7%)	4.807	**0.028**
Current treatment with statins (Missing = 220)			23 (5.2%)	10 (5.7%)	13 (4.9%)	0.139	0.709
GAF score (Missing = 284)			56.96 (±14.38)	59.76 (±12.71)	55.21 (±15.08)	3.157	**0.002**
YMRS score (Missing = 161)			20.38 (±10.51)	17.11 (±8.92)	22.22 (±10.89)	5.685	**<0.001**
HAM-D (Missing = 501)			14.58 (±6.65)	14.19 (±5.61)	14.77 (±7.11)	0.519	0.605
MADRS (Missing = 545)			22.60 (±8.65)	21.77 (±7.23)	23.06 (±9.36)	0.846	0.400
BPRS (Missing = 140)			40.89 (±9.17)	37.13 (±7.80)	42.91 (±9.22)	7.588	**<0.001**
HAM-A (Missing = 563)			8.90 (±4.35)	8.76 (±4.02)	8.98 (±4.55)	0.253	0.801

Legend: BMI = Body Mass Index; BPRS = Brief Psychiatric Rating Scale; χ^2^ = chi-square; GAD = Generalized Anxiety Disorder; GAF = Global Assessment of Functioning; HAM-A = Hamilton Anxiety Scale; HAM-D = Hamilton Depression Rating Scale; MADRS = Montgomery and Asberg Depression Rating Scale; OCD = Obsessive Compulsive Disorder; t = Student’s t; YMRS = Young Mania Rating Scale. Means for quantitative variables and frequencies for qualitative ones are reported. Standard deviations for quantitative variables and percentages for qualitative variables are reported in brackets. In bold is statistically significant *p* resulting from χ^2^ or unpaired Student’s *t* tests (*p* ≤ 0.05).

**Table 2:** Biological variables of the total sample and of the two groups divided according to the presence of lifetime psychotic symptoms.

Variables	Total Sample N = 665	−Psychotic Symptoms N = 236 (35.5%)	+Psychotic Symptoms N = 429 (64.5%)	t	*p*-Value
Number of RBC (10^12^/L) (Missing = 168)	4.60 (±0.56)	4.58 (±0.59)	4.60 (±0.54)	0.416	0.677
MCV (fL) (Missing = 310)	86.60 (±8.71)	86.26 (±9.68)	86.75 (±8.24)	0.487	0.627
HB (g/dL) (Missing = 167)	13.64 (±1.69)	13.53 (±1.73)	13.70 (±1.67)	1.050	0.294
Number of WBC (10^9^/L) (Missing = 178)	7.72 (±2.65)	7.65 (±2.71)	7.75 (±2.62)	0.371	0.771
Number of lymphocytes (10^9^/L) (Missing = 265)	2.18 (±0.75)	2.32 (±0.76)	2.11 (±0.73)	1.658	**0.008**
Number of neutrophils (10^9^/L) (Missing = 266)	4.08 (±2.49)	3.65 (±2.44)	4.28 (±2.50)	2.353	**0.019**
NLR (Missing = 266)	2.15 (±1.76)	1.79 (±1.63)	2.31 (±1.79)	2.776	**0.006**
Number of PLT (10^9^/L) (Missing = 314)	245.72 (±67.6)	249.24 (±67.55)	244.10 (±67.65)	0.663	0.508
MPV (fL) (Missing = 315)	11.02 (±4.52)	10.79 (±1.06)	11.12 (±5.40)	0.620	0.536
PCHE (U/L) (Missing = 434)	7280.05 (±1736.67)	7341.29 (±2073.18)	7253.43 (±1574.68)	0.353	0.720
Total plasmatic proteins (g/dL) (Missing = 341)	6.59 (±0.57)	6.63 (±0.57)	6.57 (±0.57)	0.849	0.396
Albumin (g/dL) (Missing = 330)	4.25 (±0.43)	4.24 (±0.42)	4.26 (±0.43)	0.304	0.761
Bilirubin (mg/dL) (Missing = 200)	0.57 (±0.41)	0.50 (±0.34)	0.60 (±0.43)	2.348	**0.019**
Uric acid (mg/dL) (Missing = 326)	5.20 (±1.92)	5.18 (±1.80)	5.21 (±1.97)	0.149	0.882
Cholesterol (mg/dL) (Missing = 220)	176.32 (±41.21)	182.81 (±40.52)	173.05 (±41.24)	2.369	**0.018**
LDL (mg/dL) (Missing = 504)	105.75 (±35.82)	109.92 (±33.87)	103.59 (±36.76)	1.064	0.289
HDL (mg/dL) (Missing = 488)	52.44 (±16.10)	51.69 (±15.75)	52.80 (±16.32)	0.430	0.668
Glycaemia (mg/dL) (Missing = 190)	94.31 (±26.73)	93.31 (±23.17)	94.79 (±28.30)	0.566	0.572
Urea (mg/dL) (Missing = 363)	29.80 (±16.63)	31.33 (±16.68)	29.14 (±16.61)	1.051	0.294
Creatinine (mg/dL) (Missing = 177)	0.85 (±0.34)	0.89 (±0.51)	0.83 (±0.23)	1.461	0.146
CPK (U/L) (Missing = 333)	215.18 (±334.41)	156.33 (±179.60)	243.17 (±384.11)	2.807	**0.005**
TSH (mcU/mL) (Missing = 299)	2.14 (±2.26)	2.25 (±1.75)	2.08 (±2.49)	0.691	0.490
PCR (mg/L) (Missing = 525)	1.32 (±2.86)	1.26 (±2.78)	1.33 (±2.89)	0.116	0.908
AST (UI/L) (Missing = 343)	26.75 (±37.20)	24.07 (±12.11)	28.09 (±44.70)	0.912	0.363
ALT (UI/L) (Missing = 269)	24.74 (±18.13)	26.69 (±21.55)	23.85 (±16.29)	1.311	0.192
GGT (U/L) (Missing = 286)	25.00 (±29.41)	31.21 (±42.01)	22.12 (±20.67)	2.249	**0.026**
LDH (mU/mL) (Missing = 422)	208.46 (±95.63)	208.73 (±76.95)	208.35 (±102.43)	0.028	0.978
Triglycerides (mg/dL) (Missing = 474)	115.77 (±73.73)	139.50 (±97.60)	104.90 (±56.94)	2.554	**0.013**
Serum iron (mcg/dL) (Missing = 458)	83.29 (±39.33)	75.51(±32.17)	86.85 (±41.83)	1.938	0.054
Sodium (mEq/L) (Missing = 305)	141.73 (±2.69)	142.05 (±2.44)	141.59 (±2.80)	0.297	0.138
Potassium (mmol/L) (Missing = 309)	4.15 (±0.41)	4.20 (±0.43)	4.13 (±0.40)	1.487	0.172
Na/K ratio (Missing = 310)	34.48 (±3.45)	34.26 (±3.33)	34.59 (±3.50)	0.835	0.404

ALT = alanine transaminases; AST = aspartate transaminase; CPK = creatine phosphokinase; GGT = gamma-glutamyl-transferase; HB = hemoglobin; HDL = high-density lipoproteins; LDH = lactate dehydrogenase; LDL = low-density lipoproteins; MCV = mean corpuscular volume; MPV = mean platelet volume; Na/K ratio = sodium/potassium ratio; NLR = neutrophil/lymphocyte ratio; PCHE = pseudocholinesterase; PCR = C-reactive protein; PLT = platelets; RBC = red blood cells; t = Student’s t; TSH = thyroid-stimulating hormone; WBC = white blood cells. Means and standard deviations (in brackets) are reported. In bold is statistically significant *p* resulting from unpaired Student’s *t* tests (*p* ≤ 0.05).

**Table 3:** Final binary logistic regression model.

Variables	B	S.E.	Wald	*p*-Value	OR	95% CI for OR
Number of manic episodes	0.063	0.052	1.481	0.224	1.065	0.962–1.179
Comorbidity with a diagnosis of personality disorder	−0.634	0.412	2.369	0.124	0.531	0.237–1.189
Duration of hospitalization	0.058	0.022	7.245	**0.007**	1.060	1.016–1.105
YMRS score	0.112	0.025	20.379	**<0.001**	1.118	1.065–1.174
NLR	0.144	0.092	2.459	0.117	1.155	0.965–1.383
Type of current episode: major depression versus mania	−1.843	0.561	10.776	**0.001**	0.158	0.053–0.476

In this analysis, the dependent variable was psychotic versus non-psychotic bipolar disorder. B = regression coefficient; CI = confidence interval; NLR = neutrophil to lymphocyte ratio; OR = odds ratio; S.E. = standard error of B; Wald = Wald statistics; YMRS = Young Mania Rating Scale. In bold is statistically significant *p* (≤0.05).

## Data Availability

Data are available under request to the corresponding author.
